# Factors affecting the number of road traffic accidents in Kerman province, southeastern Iran (2015–2021)

**DOI:** 10.1038/s41598-023-33571-8

**Published:** 2023-04-24

**Authors:** Mohsen Behzadi Goodari, Hamid Sharifi, Paria Dehesh, Mohammad Amin Mosleh-Shirazi, Tania Dehesh

**Affiliations:** 1 Department of Biostatistics and Epidemiology, School of Public Health, Kerman University of Medical Sciences, Kerman, Iran; 2 HIV/STI Surveillance Research Center, and WHO Collaborating Center for HIV Surveillance, Institute for Futures Studies in Health, Kerman University of Medical Sciences, Kerman, Iran; 3 Department of Epidemiology, School of Public Health, Iran University of Medical Sciences, Tehran, Iran; 4 Ionizing and Non-Ionizing Radiation Protection Research Center (INIRPRC), School of Paramedical Sciences, Shiraz University of Medical Sciences, Shiraz, Iran; 5 Modeling in Health Research Center, Institute for Futures Studied in Health, Kerman University of Medical Sciences, Kermen, Iran

**Keywords:** Ecology, Health policy

## Abstract

Road traffic accidents (RTAs) are among the top causes of mortality and disability globally, particularly in developing nations like Iran. In this study, RTAs were analyzed to develop precise predictive models for predicting the frequency of accidents in the Kerman Province (southeastern Iran) using the autoregressive integrated moving average (ARIMA) and autoregressive integrated moving average with explanatory variables (ARIMAX) modeling methods. The findings demonstrate that including factors regarding humans, vehicles, and elements of nature in the time-series analysis of accident records resulted in the development of a more reliable prediction model than utilizing only aggregated accident count. The understanding of safety on the road is increased by this research, which also offers a method for forecasting that utilizes a variety of parameters relating to people, cars, and the environment. The findings of this research are likely to contribute to lowering the incidence of RTAs in Iran.

## Introduction

One of the major issues facing the globe today is the number of accidents and fatalities on the roadways. Road accidents claim the lives of 1.24 million people annually, injure 20–50 million more, and cost more than $500 billion globally^1^. The value of traffic injuries in low- and middle-income nations is reported to be 1–2% of the GDP, or approximately US $100 billion a year^2^. Ninety percent of these fatalities occur in low- and middle-income countries, according to current trends, and if sufficient action is not taken, road traffic injuries may become the seventh major cause of death by 2030^3^. Along with the psychological anguish of losing a loved one, an RTA has an adverse psychological impact on accident victims and makes them permanently disabled. RTAs have several underlying causes and are influenced by the interactions of a number of pre-accident variables, including people, cars, and the surrounding environment^4^.

In order to identify and comprehend the reasons behind causing RTAs and take appropriate actions, much research has been conducted. In a study by Liang et al.^5^, using logistic regression analysis, it was shown that speeding, driving while drunk, and driving while tired was substantially related to road accidents in Suzhou, Anhui Province, China. Another study by Bakhtiyari et al.^6^ demonstrated that factors contributing to the rising accident rate on Iran's significant roadways included careless overtaking, excessive speeding, weariness, and failure to pay attention to the forward direction. It is also well recognized that meteorological elements like extreme temperature and humidity raise the risk of having a motor vehicle accident^6–8^.

In industrialized nations, the majority of research on RTAs have produced results that have been useful in creating countermeasures which have consequently reduced RTAs in those countries^9^. The situation, however, has not been the same in all developing countries, particularly Iran, which is listed as one of the countries with the greatest number of deaths and injuries associated to RTAs^10^. Unsafe road vehicles, poor road design, unsuccessful compliance with road safety laws, and insufficient prediction models for road accidents taking into account factors contributing to an accident have all contributed to this alarming rate of RTAs in this country^10^. It is thus required to conduct a more thorough investigation of traffic accidents in order to design policies and initiatives that would minimize road accidents in Iran and bring the country closer to reaching the goals of the United Nations' ‘decade of action on road safety (2011–2020)’.

For the purpose of proposing suitable preventive measures or addressing the contributing factors in Iran, it is crucial to comprehend how traffic accidents will develop over time. It is possible to verify the relevance of certain variables using time series analysis, an effective method for generating future predictions^11^.

The Box and Jenkins autoregressive integrated moving average (ARIMA) model is the most often used time series analysis method in research on road safety^12^. For combined time series count data, Quddus suggested that the ARIMA model is the best accident-predicting model^13^. Most of the research utilizing ARIMA models just evaluated the overall number of accidents without taking into consideration the influencing factors that impacted accident occurrences^14^. However, a number of studies have examined unrelated variables that occurred within the same time period to assess any association between the number or intensity of accidents and explanatory variables^15,16^.

While certain influencing elements have been considered in some studies regarding developed countries, very little has been published on examination of all driver factors contributing to traffic accidents. Further research is needed to examine and forecast RTAs in developing countries, taking a broad range of factors related to people, vehicles, and the environment into account. Iran is one of the largest nations in the Middle East, where accidental deaths are among the leading causes of mortality. Planning to control and minimize the fatality rate related to accidents can be successful when considering the combined effects of environmental, human, and vehicle factors.

The goal of the present research was to develop an effective time series accident forecasting model that takes into account a variety of individual, vehicle, and environmental (road) predictors, as opposed to only relying on an aggregated accident count. Accuracy metrics such as the Akaike Information Criteria (AIC), Akaike Information Criteria (AICc), corrected AICc, and Bayesian Information Criteria (BIC) were used in this study to evaluate how well the univariate time series model (ARIMA model) performed when combined with the multiple time series model (ARIMAX model). The findings will help in choosing the model that works best.

## Methods

### Design

In this secondary data analysis, the existing data were analyzed. In this study, all RTAs that occurred in Kerman province from March 20, 2015, to March 20, 2021, were collected monthly and based on the summary report from the police accident center. These forms consist of data including the type, severity, time and causes of the accident, and the name and type of the vehicle at fault. Meteorological data such as average relative humidity and average monthly temperature were also obtained from Kerman Meteorological Department. In this study, the response variable is the number of accidents per month for 6 years and a total of 72 data items. The analysis used a monthly count record that included minor, major, and fatal incidents.

### Analysis plan

The collected data was investigated in the R software environment using the ARIMA and ARIMAX models. The Box Box-Cox and Dickey-Fuller tests were used to determine if a unit root was present in the accident time series.

#### Autoregressive integrated moving average (ARIMA) model

The ARIMA models are among the best time series analytic techniques for the examination of autocorrelated data. In this model, the MA (q) parameter displays the linear relationship between the time series' present value and its past and current residual values. The AR (p) parameter displays the linear relationship between the time series' most recent value and its prior values as well as its most recent residuals. The generic version of its formula is the following:

1 $$\Delta d{\text{y}}_{{\text{t}}} = \left( {\upalpha \left( {\text{A}} \right)} \right)/(\upvarphi \left( {\text{A}} \right))\upvarepsilon_{{\text{t}}}$$

where *y*_*t*_ is the outcome series (monthly count of accidents), *t* is order of time, *ε*_*t*_ is the random error (white noise) at time of *t*, *A* is operating the backshift, $\Delta$ is the integrated processes (where $\Delta d$ y_t_ = y_*t*_ − y_t_ − 1), the non-seasonal difference of order *d* is required to guarantee the stationary features of time serie*s,* and the extra parameters of the model are defined as follows:

2 $$\upalpha \left( {\text{A}} \right) = \left( {{1} - \, \uptheta_{{1}} {\text{A }} - \, \uptheta_{{2}} {\text{A}}^{{2}} - \, \cdots \, - \, \uptheta_{{\text{q}}} {\text{A}}^{{\text{q}}} } \right)$$

3 $$\upvarphi \left( {\text{A}} \right) \, = \, \left( {{1} - \, \upvarphi_{{1}} {\text{A }} - \, \upvarphi_{{2}} {\text{A}}^{{2}} - \, \cdots \, - \, \upvarphi_{{\text{p}}} {\text{A}}^{{\text{p}}} } \right)$$

where … *φ*_*1*_*, φ*_*2*_*, … φ*_*p*_ are the autoregressive (AR) parameters, θ _*1*_*,* θ _*2*_*, …* θ _*q*_ are the moving average (MA) parameters, *p* is the order of autoregressive part, and *q* is the order of the moving average part.

Three key phases are used to determine the most efficient model: model development, parameter estimations, and diagnostic verification^17^.

#### Model development

Identifying if the time series is stationary is the first stage in creating an ARIMA model. When the statistical characteristics of a time series—such as its mean, variance, or autocorrelation—remain consistent across time, it is considered to be stationary. The enhanced Box-Cox and Dickey-Fuller tests and the plot of autocorrelation functions were utilized to examine the stationary or accident time series data used in this research.

The Box-Cox Transformation method is used to stabilize the series variance. The augmented Box-Cox Transformation equation is as presented in Eq. (4):

4 $${\text{ yt}}\left( {\uplambda } \right){ } = \left\{ {\begin{array}{*{20}c} {\frac{{y_{t}^{\lambda } - 1}}{\lambda },} & {\lambda \ne 0} \\ {\log y,} & {\lambda = 0} \\ \end{array} } \right.$$

where λ is the power coefficient of the transformation that is needed to be selected; When λ = 1, the whole series is identical only with a shift; When λ = 0, the transformation is the logarithm.

Equation (5) presents the enhanced Dickey-Fuller regression equation:

5 $${\text{y}}_{{\text{t}}} = \, \upalpha \, + \, \uprho {\text{y}}_{{{\text{t}} - {1}}} + \sum\limits_{i = 1}^{k} {\upphi_{i} \Delta y_{{{\text{t}} - 1}} + \upbeta t + \upvarepsilon_{{\text{t}}} }$$

where *y*_*t*_ is the outcome variable (count of accidents), Δ*y*_*t-i*_ is the time-lagged variation in the outcome variable, *ε*_*t*_ is the white noise error term, and *t* is order of time. The null hypothesis—that the series has a unit root—cannot be rejected if the P-value in the augmented Dickey-Fuller test is greater than the significance alpha value. Finding the differences is the primary method for making a non-stationary series with a unit root stationary. The number of the moving average (MA) and autoregressive (AR) components are determined using the autocorrelation function (ACF) and partial autocorrelation function (PACF).

#### Model parameter estimation

The parameters of the selected model were estimated using the maximum likelihood method, and the statistical significance of the model was evaluated using P-values. The best model among the different ARIMA models generated in the current research was chosen using the lowest Bayesian information criterion (BIC), lowest Akaike information criterion (AIC), and lowest Akaike Information Criteria, modified (AICc). The BIC is written as:

6 $$BIC \, = \, n.ln\left( {RSS/n} \right) \, + \, k.ln\left( n \right)$$

In this formula, *n* stands for the count of useful data used to fit the model, *k* for the count of model parameters, and *RSS* for residual sum of squares. The AIC is expressed as:

7 $${\text{AIC }} = {\text{ 2k }}{-}{\text{ 2ln}}({\text{L}})$$

where *k* refers to the count of parameters in the model and L refers to the maximum likelihood function.

The AICc is expressed as in Eq. (8).

8 $${\text{AICc }} = {\text{ AIC }} + \frac{{2k^{2} + 2k}}{n - k - 1}$$

#### Model diagnostic checking

The residuals ACF and PACF, as well as the Shapiro–Wilk test, Shapiro–Wilks plot, and Q.Q. plot, were used to evaluate the model's suitability in light of the residuals' attributes.

#### Autoregressive integrated moving average model with explanatory variables (ARIMAX) model

The pure ARIMA model may be logically extended to include independent variables, which give the model more explanatory power. This is what the ARIMAX model does. It is also called a transfer function model^18^. The single-output, multiple-input transfer function model used in this research is presented in Eq. (9). It was created by adding non-stationary input series (explanatory variables) to Eq. (1).

9 $$\nabla^{{\text{d}}} {\text{y}}_{{\text{t}}} = \sum\limits_{k = 1}^{k} {{\text{v}}_{{\text{k}}} \left( {\text{B}} \right)\nabla^{{\text{d}}} {\text{X}}_{{{\text{k}},{\text{t}}}} + \, \left( {\upalpha \left( {\text{A}} \right)} \right)/\left( {\upvarphi \left( {\text{A}} \right)} \right) \, \upvarepsilon {\text{t}}}$$

where (α(A))/(ϕ(A)) εt is the system's noise series, which is considered to be autonomous of the explanatory variables, X_k,t_ stands for the accident explanatory variables, and Vi (B) is the transfer function for the kth input series, and which may be represented as;

10 $${\text{V}}\left( {\text{B}} \right) \, = \frac{{w(B)B^{b} }}{\delta (B)}$$

where the numerator can be expanded to *ω*(*B*) = *ω*_*0*_ – *ω*_1_* B* − … − *ω*_*s*_*B*^*s*^, and the denominator can be expanded to *δ*(*B*) = 1- *δ*_*1*_*B* − … − *δ*_*r*_*B*^*r*^, *s,* and *r* stand for the polynomial's orders, while *b* is the delay parameter, which denotes the real interval of time before the input series has an impact on the output series. The terms in transfer functions all vary by the same amount. For time series with non-stationary input and output:

11 $$\nabla^{{\text{d}}} {\text{y}}_{{\text{t}}} = \sum\limits_{k = 1}^{k} {\frac{{\omega_{k} (B)B^{bk} }}{{\delta_{k} (B)}}\nabla_{{\text{t}}} {\text{X}}_{{{\text{k}},{\text{t}}}} + \, \left( {\upalpha \left( {\text{A}} \right)} \right)/\left( {\upvarphi \, \left( {\text{A}} \right)} \right) \, \upvarepsilon {\text{t}}}$$

Similar to how a univariate Box-Jenkins ARIMA model is created, the ARIMAX (transfer function model) is created via an iterative process that involves model identification, parameter estimates, and diagnostic testing.

#### Identification

Determination of the transfer function involves prewhitening both the output and the input series, computing the cross-correlation functions of the prewhitened series, and finding out the order of *b*, *s*, and *r*. Prewhitening was achieved by fitting an ARIMA model to each input series so that the residuals were reduced to white noise, followed by filtering the input series using the model to produce white noise series. To get residual series of white noise from the output series, the same ARIMA model was used. The process of prewhitening for non-stationary series is given by:

12 $$\nabla^{{\text{d}}} {\text{X}}_{{{\text{it}}}} = \frac{{\uptheta_{x} (B)}}{{\upvarphi_{x} (B)}}e_{it}$$

13 $${\text{E}}_{{{\text{it}}}} = \frac{{\upvarphi_{x} (B)}}{{\uptheta_{x} (B)}}\nabla^{{\text{d}}} {\text{X}}_{{{\text{it}}}}$$

14 $$\beta_{{{\text{it}}}} = \frac{{\upvarphi_{x} (B)}}{{\uptheta_{x} (B)}}\nabla^{{\text{d}}} {\text{y}}_{{{\text{it}}}}$$

where *e*_*it*_ and *β*_*it*_ refer to white noise series with mean, zero, and variance,* σ*^2^.

Various lags, L (L = 0, 1, 2, …), were used to assess the cross-correlation functions between the prewhitened input and output series. The estimated values were then contrasted with theoretical impulse response functions of various orders to get a general understanding of the delay parameter *b* and the orders of *r* and *s* of the transfer function between the output and input series^18^.

### Ethics information

This project was approved by the Research Ethics Committee of Kerman University of Medical Sciences (Ethics code: IR.KMU.REC. 1397/352). The Helsinki Declaration was followed in the conduct of this investigation. Individuals’ data were not used in this investigation, hence informed permission or a formal ethics review were not necessary.

## Results

In this section, the outcomes of various models are shown.

### Results of ARIMA and ARIMAX models

**Figure 1:**
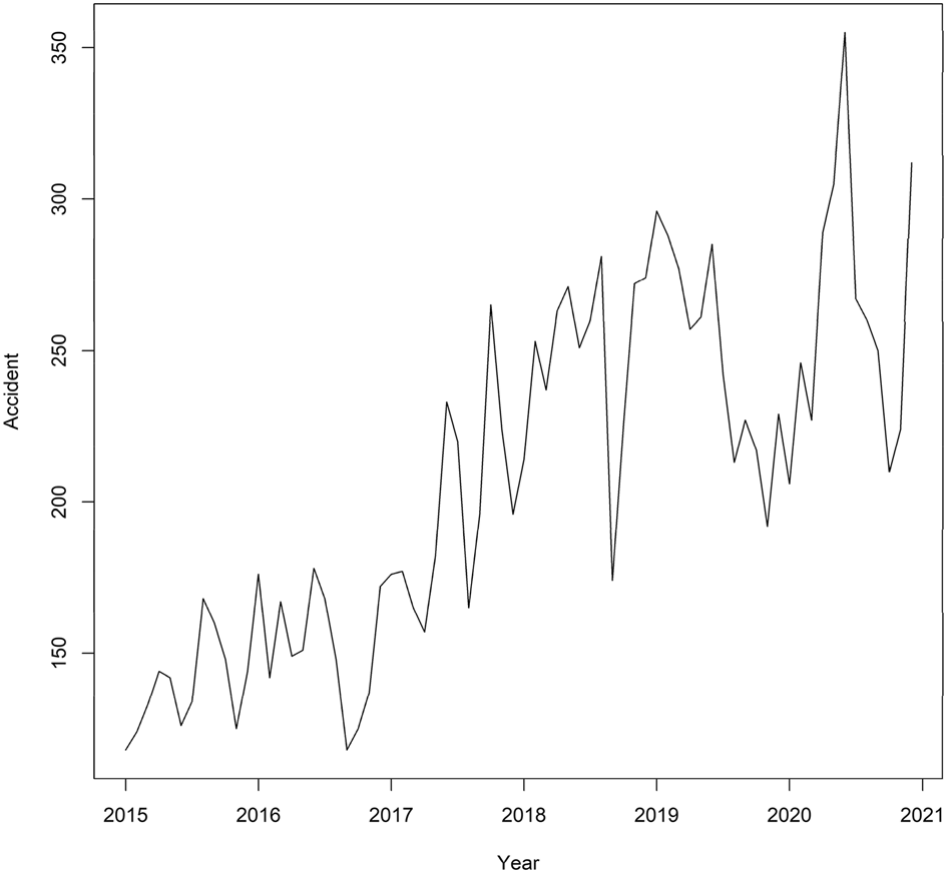
Time series plot of the number of accidents in Kerman province, Iran.

The time series plot of the monthly count data for the total number of accidents is shown in Fig. 1. Accidents due to inattention to the forward direction, loss of control, brake failure, unauthorized speed, accidents with heavy vehicles, passenger cars, motorcycles, and pickup cars, average humidity, and average temperature are among the contributing factors to the accidents, which were applied in the current investigation and examined during the same time period.

The outcome variable and the explanatory factors' descriptive statistics are shown in Table 1.

**Table 1:** Descriptive statistics of RTA data.

Variable	Maximum	Minimum	Mean	Std. deviation
Outcome variable				
Number of accidents	1428	1125	1247	82.38
Explanatory variables				
Inattention to forward direction	299	140	267.83	45.52
Loss of control	659	521	587.58	49.49
Brake failure	769	569	659.41	62.63
Unauthorized speed	189	150	165.16	10.77
Accidents with passenger cars	820	639	718.25	51.74
Accidents with heavy vehicles	178	111	131.67	22.38
Accidents with pickup cars	198	127	160.16	21.74
Accidents with motorcycles	269	129	196.41	46.12
Average temperature (°C)	32.02	8.9	20.61	8.6
Average humidity (%)	43.45	17.14	30.08	10.16

As shown in Fig. 1, there is a regular variation in the number of accidents, which points to an increasing tendency in the data. The findings of the Box-Cox test showed stable data variance (= 0.357, P = 0.431). The sample ACF plot further revealed that the series were not stable in mean since the autocorrelation coefficients at different lags did not fall within the confidence range ("Supplementary Fig. S1"). The upgraded Dickey–Fuller test (Z = 0.441) demonstrated that the series were non-stationary since the estimated P-value (P = 0.66) was greater than the significant alpha threshold (= 0.05). The test could not find evidence to support the idea that the series had no unit root. This suggests that for the time series to become stationary, at least the first difference must occur.

The time series plot of the first non-seasonal variation in the number of accidents is shown in Fig. S2 in the supplemental material. The first-order non-seasoning differencing strategy was used to eliminate the trend from the time series, as seen in this figure (which was stationary in the mean). As shown in Fig. S3a in the supplementary data, the first difference of the time series plot of the ACF decays very fast, proving that the first-order non-seasonal difference of the series was sufficient to render the time series stationary. This figure also displays the partial autocorrelation function's initial difference, which indicates the existence of an autoregressive component in the time series. The enhanced Dickey-Fuller test (Z = − 9.405) also supported the stationarity of the initial difference of the time series since the resultant P-value (0.001) is smaller than the significant alpha criterion (0.05).

Determining the correct ARIMA model for the number of accidents from Fig. S3 was not easy. It was difficult to determine the appropriate ARIMA model for the amount of incidents from Fig. S3. BIC, AIC, and AICc were used to assess several ARIMA models in order to identify the model that best matched the RTA data and choose the model with the best fit. The model with the lowest BIC, AIC, and AICc among the possibly significant models was selected. If every aspect of Model A is significant at a 95% confidence level, it is deemed to be significant. Table 2 compares several criteria for selecting the best ARIMA model. The ARIMA(0, 1, 2) was selected as the best model out of those being considered since it has the lowest BIC (699.66) and AIC (693.24), as indicated in the table, and all of its parameters were significant. The created ARIMA(0, 1, 2) model is sufficient to characterize the frequency of incidents in the province, per the model fit data in Table 3.

**Table 2:** Summary of estimation for ARIMA models.

ARIMA(p,d,q)	BIC	AIC	AICc
ARIMA(1,1,1)	700.71	694.28	− 343.96
ARIMA(1,1,2)	703.74	695.29	− 343.34
ARIMA(2,1,1)	703.81	695.37	694.76
ARIMA(0,1,1)	701.6	697.25	697.08
ARIMA(0,1,3)	703.69	695.23	− 343.31
*ARIMA(0,1,2)*	*699.66*	*693.24*	− *343.34*
ARIMA(3,1,0)	705.93	697.48	− 344.42
ARIMA(3,1,1)	707.88	697.49	− 343.29
ARIMA(2,1,2)	707.95	697.56	696.63

Significant values are in Italics.

**Table 3:** The coefficients of the ARIMA(0,1,2) model for the number of road accidents in Kerman province (2015–2021).

Variable	Coefficient	Standard deviation	P-value	Confidence interval
constant	2.24	0.14	< 0.001	(1.98, 3.12)
MA1	− 0.34	0.11	< 0.001	(− 0.63, − 0.19)
MA2	− 0.29	0.11	< 0.004	(− 0.62, − 0.12)

Table 3 shows the significant coefficients of the ARIMA(0, 1, 2) model.

**Figure 2:**
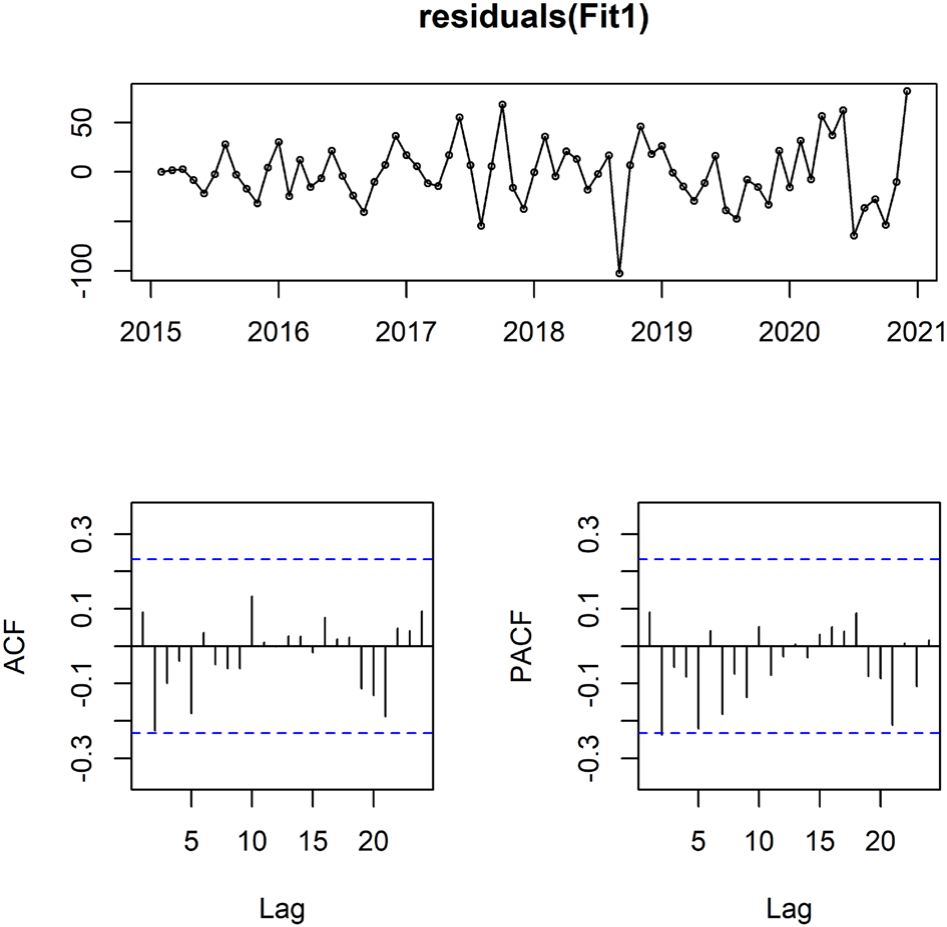
The general trend of the residuals series with ACF and PACF plots.

Figure 2 shows the general trend of the residuals series with ACF and PACF plots.

**Figure 3:**
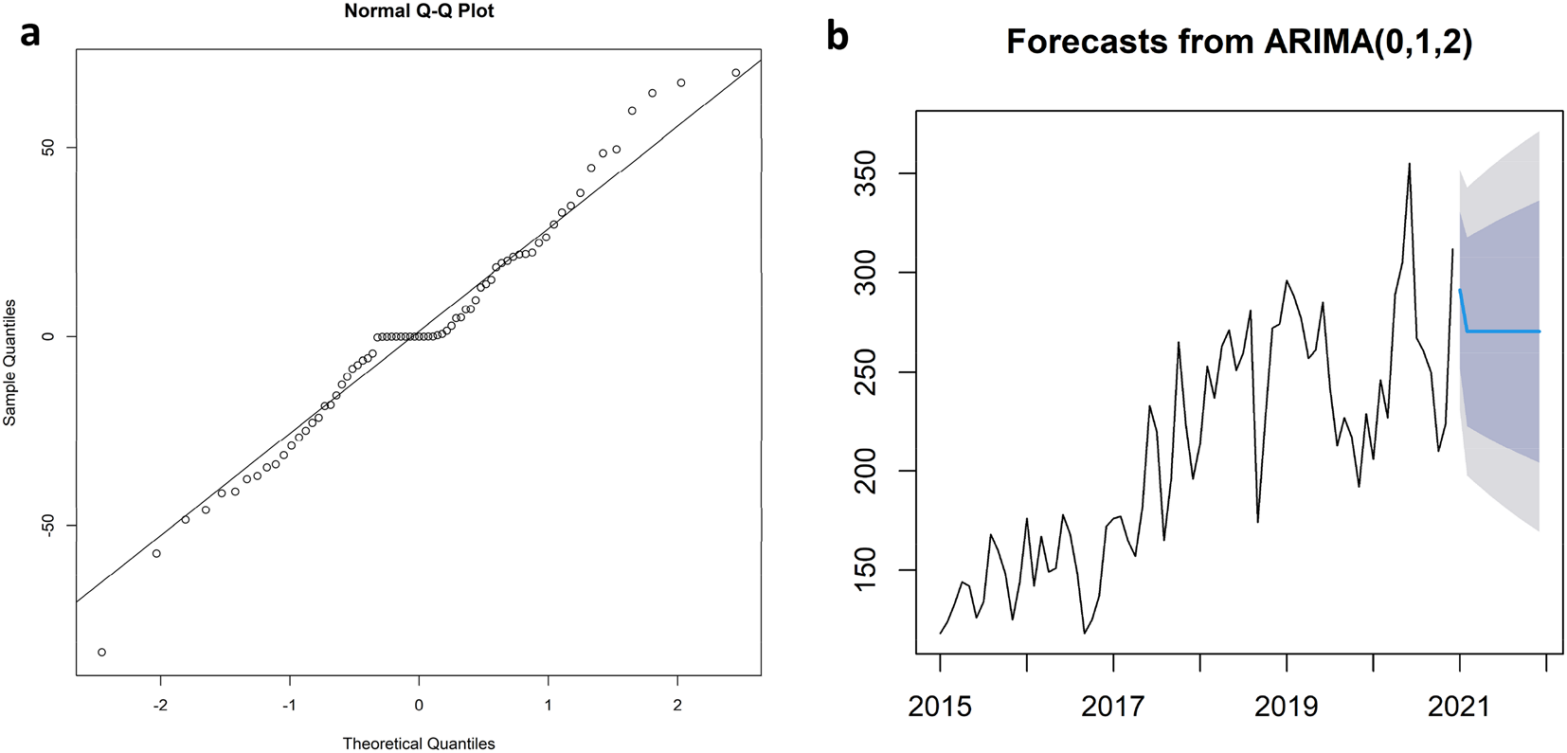
(**a**) Residuals Q.Q. plot, (**b**) Model prediction plot.

The model is suitable for time series forecasting of RTA in Kerman province, as shown by the Shapiro–Wilk test findings (W = 0.978, P = 0.254) to normal distribution of residuals and the diagnostic check to validate the ARIMA(0,1,2) model suitability. Another proof of the residuals' normalcy is provided by the Q.Q. plot, which also displays all the residuals around the line at a 45-degree angle (Fig. 3a). The range of model predictions is shown in Fig. 3b.

Additionally, same actions were taken for variables (explanatory variables mentioned in Table 1) the data of which showed a trend over time. Each independent variable entered the original ARIMA model of the response variable to fit the ARIMAX model with the same delays after being seen to have a substantial influence on the outcome variables at various delays (Table 4).

**Table 4:** Models related to independent variables, along with coefficients and goodness of fit criteria.

Explanatory variables	Model	AR1	MA1	MA2	SAR1	SMA1	BIC	AIC	AICc
Loss of control	(0,1,2)	–	− 0.27	− 0.47	–	–	614.21	607.78	607.42
Brake failure	(1,0,1)	0.94	− 0.44	–	–	–	653.99	645.48	644.89
Accidents with Passenger cars	(1,0,1)	0.97	− 0.62	–	–	–	587.49	578.38	578.98
Accidents with heavy vehicles	(1,0,1)	0.91	− 0.67	–	–	–	125.92	116.81	117.41
Accidents with pickup cars	(1,0,2)	0.97	− 0.41	− 0.28	–	–	119.87	108.49	109.42
Accidents with motorcycles	(1,0,0)	0.54	–	–	–	–	530.63	523.81	524.15
Inattention to forward direction	(1,0,0)	0.75	–	–	–	–	538.92	532.44	532.09
Unauthorized speed	(1,0,1)	− 0.61	− 0.62	–	–	–	475.96	467.45	466.85
Average humidity	(1,0,1)	0.99	− 0.96	–	–	− 0.92	− 378.57	− 386.95	− 386.22
Average temperature	(0,1,1)	–	− 0.44	–	–	− 0.61	230.17	223.94	224.38

*AR1* first order autoregressive, *MA1* first order moving average, *MA2* second order moving average, *SAR1* first order seasonal autoregressive, *SMA1* first order seasonal moving average, *BIC* Bayesian information criteria, *AIC* Akaike information criteria, *AICc* Akaike information criteria, corrected.

Table 5 shows inattention to forward direction, unauthorized speed, brake failure, and average temperature (all with a lag of one month) with the largest significant coefficients among the most important explanatory variables for estimating the number of road accidents.

**Table 5:** The coefficient for the ARIMAX model with independent loss of control, brake failure, passenger cars, heavy vehicles, pickup cars, motorcycles, inattention to forward direction, unauthorized speed, average humidity, and average temperature variables.

Explanatory variables with relevant lags	Coefficient	Standard deviation	P-value	Confidence interval
1 ~ Loss of control	0.43	0.05	0.042	(0.23, 0.63)
1 ~ Brake failure	0.69	0.01	0.002	(0.44, 0.91)
2 ~ Accidents with passenger cars	0.54	0.03	0.031	(0.38, 0.72)
1 ~ Accidents with heavy vehicles	0.37	0.09	0.043	(0.21, 0.68)
1 ~ Accidents with pickup cars	0.24	0.23	0.051	(− 0.43, 0.44)
1 ~ Accidents with motorcycle	0.03	0.06	0.133	(− 0.52, 0.82)
4 ~ Accidents with motorcycle	0.12	0.08	0.112	(− 0.67, 0.73)
1 ~ Inattention to forward direction	0.82	0.01	0.014	(0.02, 0.92)
1 ~ Unauthorized speed	0.81	0.02	0.001	(0.03, 0.97)
4 ~ Average humidity	0.38	0.02	0.048	(0.22, 0.82)
1 ~ Average temperature	0.62	0.001	0.003	(0.24, 0.74)
MA1	− 0.76	0.09	< 0.001	(− 1.22, − 0.43)
MA2	− 0.28	0.12	< 0.001	(− 1.14, − 0.11)

~ : Shows influential Lags of explanatory variables on the response.

The positive coefficients of all the explanatory variables, which are all statistically different from zero, show that an improvement in any of the contributing factors will result in an increase in the number of accidents. Figure S4 displays a graph of the total number of accidents predicted by the final ARIMAX model (dotted line) and a list of actual incidents (dashed line). It is evident that the model's predicted values and actual values agree extremely well.

## Discussion

According to the study's findings, the factors of inattention, excessive speed, vehicle rollover and brake failure accidents, average temperature and humidity, and accidents involving passenger vehicles and pickup cars are the key predictors of the frequency of accidents. Inattention was one of the most important elements in this research that affected the frequency of accidents. This is consistent with the findings of other studies^17^ and may be caused by elements like exhaustion and drowsiness, work pressure, talking with a passenger while driving, and the overall number of driving hours^19^.

Kerman is the largest province of Iran, with an area of 186,422 km^2^, located in the southeastern part of Iran. The main transit road between Bandar Abbas (the main port of Iran) and the center of Iran with a high traffic load passes through Kerman. This province has different weather conditions (desert to cold and mountainous) and could be a good pattern for other provinces. The drivers experience different weather conditions when crossing Kerman roads. For example, the road from Bandar Abbas to Kerman has humid weather in the mountains and is very cold in the desert. This was the reason for choosing Kerman province for this research.

Unauthorized speed was one of the most significant factors in the current research that affected the frequency of accidents. Unauthorized speed was listed among the top risk factors for accidents in the earlier studies' findings^6,8,20^. This could be due to the lack of speed cameras on various roads and the lack of imposition of fines. A study conducted in Spain over eight years and two stages (before and after intervention and application of a penalty point system) to prevent traffic violations, including unauthorized speed showed that after the intervention, the number of accidents and accident injuries were significantly reduced^21^.

Another influential factor on the study's accident count was the average temperature. Gao et al. in Shantou, China, and Yannis and Karlaftis in Athens both reported that accidents happen more often at higher temperatures^22^. On hot days, high temperatures can cause drivers to become distracted, make more mistakes while driving, and become tired and drowsy. Temperatures below zero can also lead to slippery road surfaces, and as a result, increase the number of accidents^23,24^. In contrast, the study of Meshari AI-Harbi et al. in Kuwait showed that the number of accidents decreased with increasing temperature, which is inconsistent with the results of this study. The decline was ascribed to the drop in traffic volume brought on by the yearly shutdown of educational institutions and personnel^25^.

The results of this study show that average humidity is one of the variables affecting the number of accidents. The findings of research by Satterthwaite in California, Eshghi, et al. in Ardabil, Iran, and Dhont in Vietnam are in agreement with this one^26,27^. Other research suggests that a combination of high temperatures, low air pressure, moderate-to-high humidity in China, and high humidity in winter in Kuwait are significant risk factors for traffic accidents^28^.

According to the findings of the current research, accidents resulting from brake failure affect the frequency of traffic accidents, which is similar with those of other studies^29^. Unauthorized speed, the age and gender of the drivers, and a driving history of less than two years may all contribute to this outcome^30^. The evidence shows that the proper operation of the brake system is one of the most effective ways to reduce brake failure^31^.

Another factor impacting the frequency of accidents in the analysis of the current research was a car rollover-related accident, which is similar with the findings of the study by Kattak et al.^32^. Driver error, excessive speed, drug and alcohol use, U-turns to the left or right, slippery road surfaces, brake failure, the narrow width of roads, horizontal curvature of roads, and tire bursts are among the most significant and important factors associated with car rollover, according to the studies conducted in this regard^33,34^.

Accidents between passenger cars and heavy vehicles were one of the factors in this research that affected the total number of accidents. Accidents between passenger cars and heavy vehicles have been identified as the primary cause of accidents in several studies^35–37^. This may be due to the low standards, poor quality, and rapid wear and tear of these vehicles.

In the majority of the nations throughout the globe, accidents are one of the main causes of mortality. Consequently, up-to-date research in this area is constantly required. Culture and technology are two dynamic elements that change throughout time. Road accidents are significantly impacted by these variables. Therefore, the present actions should not be based on earlier findings. By collecting more thorough information, it is vital to study and identify additional variables influencing the incidence of traffic accidents. For instance, the influence of the extent and quality of driving training and public education are of particular interest.

## Conclusion

The findings of this study suggest that the most significant elements that contribute to a rise in traffic accidents in Kerman province include distraction, speeding, brake failure, and average temperature. Therefore, training drivers and paying greater attention to these variables by installing billboards and using effective advertising techniques promise to reduce the incidence of traffic accidents.

## Supplementary Information

Supplementary Information.

## Data Availability

The data that support the findings of this study are available from the corresponding author, but restrictions apply to the availability of these data, which were used under license for the current study, and so are not publicly available. Data are however available from the authors upon reasonable request and with permission of the corresponding author.

## References

[1] Organization, W.H.O, *Global Ststus Report on Road Safety 2013* (2013).

[2] Jacobs, G., Aeron-Thomas, A., & Astrop, A. *Estimating global road fatalities *(2000).

[3] Organization, W.H., *Global status report on road safety 2015*. 2015: World Health Organization.

[4] Haddon W (1980). Advances in the epidemiology of injuries as a basis for public policy. Public Health Rep..

[5] Liang M (2020). Epidemiology of fatal crashes in an underdeveloped city for the decade 2008–2017. Int. J. Inj. Contr. Saf. Promot..

[6] Bakhtiyari M (2019). Estimating the avoidable burden and population attributable fraction of human risk factors of road traffic injuries in iran: application of penalization, bias reduction and sparse data analysis. Int. J. Inj. Contr. Saf. Promot..

[7] Shahbazi F (2019). Socioeconomic inequality in mortality from road traffic accident in Iran. J. Res. Health Sci..

[8] Dehesh T, Mardani-Fard HA, Dehesh P (2022). Forecasting of COVID-19 confirmed cases in different countries with ARIMA models. Rom. J. Diab. Nutr. Metab. Dis..

[9] Abdel-Aty M (2003). Analysis of driver injury severity levels at multiple locations using ordered probit models. J. Saf. Res..

[10] Sadeghi-Bazargani H (2016). Epidemiological patterns of road traffic crashes during the last two decades in Iran: A review of the literature from 1996 to 2014. Arch. Trauma Res..

[11] McLeod AI, Vingilis ER (2008). Power computations in time series analyses for traffic safety interventions. Accid. Anal. Prev..

[12] Box, G.E., et al., *Time series analysis: forecasting and control*. 2015: John Wiley & Sons.

[13] Quddus MA (2008). Time series count data models: An empirical application to traffic accidents. Accid. Anal. Prev..

[14] Adu-Poku K, Avuglah R, Harris E (2014). Modeling road traffic fatality cases in Ghana. Math. Theory Model..

[15] Bergel-Hayat R (2013). Explaining the road accident risk: Weather effects. Accid. Anal. Prev..

[16] Theofilatos A, Yannis G (2014). A review of the effect of traffic and weather characteristics on road safety. Accid. Anal. Prev..

[17] Box GE, Jenkins GM (1976). Time series analysis: Forecasting and control San Francisco.

[18] Chuang, A., *Time series analysis: univariate and multivariate methods*. 1991, Taylor & Francis.

[19] Craft, R.H., & Preslopsky, B. *Driver distraction and inattention in the USA large truck and national motor vehicle crash causation studies*. in *1st International Conference on Driver Distraction and Inattention (DDI 2009) Chalmers University of Technology, SwedenSAFER Vehicle and Traffic Safety CentreINRETS-ARCUEIL, FRANCE* (2009).

[20] Brenac T (2015). Influence of travelling speed on the risk of injury accident: a matched case-control study. Period. Polytech. Transp. Eng..

[21] Novoa AM (2010). Impact of the penalty points system on road traffic injuries in Spain: A time-series study. Am. J. Public Health.

[22] Gao J (2016). The association between meteorological factors and road traffic injuries: A case analysis from Shantou city China. Sci. Rep..

[23] Basagaña X (2015). High ambient temperatures and risk of motor vehicle crashes in Catalonia, Spain (2000–2011): A time-series analysis. Environ. Health Perspect..

[24] Lee W-K (2014). A time series study on the effects of cold temperature on road traffic injuries in Seoul Korea. Environ. Res..

[25] Al-Harbi M, Yassin MF, Shams MB (2012). Stochastic modeling of the impact of meteorological conditions on road traffic accidents. Stoch. Env. Res. Risk Assess..

[26] Eshghi Ali AS, Ali J, Ali N (2017). analysis of road accident dispersion leading to death aclimatic approch. Sci. Res. Q. Geogr. Data.

[27] Dhondt S (2011). Environmental health impacts of mobility and transport in Hai Phong Vietnam. Stoch. Environ. Res. Risk Assess..

[28] Song X (2019). The effect of meteorological factors on road traffic injuries in Beijing. Appl. Ecol. Environ. Res..

[29] Prentkovskis O, Sokolovskij E, Bartulis V (2010). Investigating traffic accidents: A collision of two motor vehicles. Transport.

[30] Paravar M (2013). Pre-hospital trauma care in road traffic accidents in Kashan, Iran. Arch. Trauma Res..

[31] Gargoum SA, El-Basyouny K (2016). Exploring the association between speed and safety: A path analysis approach. Accid. Anal. Prev..

[32] Khattak AJ, Schneider RJ, Targa F (2003). Risk factors in large truck rollovers and injury severity: Analysis of single-vehicle collisions. Transp. Res. Rec..

[33] Freeman, M.D., & Leith, W.M. An epidemiologic analysis of causal factors in tire failure-related traffic crashes. SAE Technical Paper (2018).

[34] Hosseinpour M (2016). Evaluating the effects of road geometry, environment, and traffic volume on rollover crashes. Transport.

[35] Rahmani K, HashemiNazari S, Ghadirzadeh M (2016). Trend analysis of traffic accidents deaths in Iran during 2006–2012: Hospital or pre-hospital occurred deaths. J. Rafsanjan Univ. Med. Sci..

[36] Khajahsalimi M, Khabiri MM, Fallah Nezhad MS (2019). Prediction and investigation of road traffic accident severity factors using support vector machine algorithm. J. Civ. Environ. Eng..

[37] Liu G (2018). Risk factors for extremely serious road accidents: Results from national road accident statistical annual report of China. PLoS ONE.

