# Supplementary material for: Factors affecting the number of road traffic accidents in Kerman province, southeastern Iran (2015–2021)
Source: Sci Rep. 2023 Apr 24;13:6662. doi: 10.1038/s41598-023-33571-8 (PMC10125984; doi:10.1038/s41598-023-33571-8)
Supplement: Supplementary file 1 — Supplementary Information. [file 41598_2023_33571_MOESM1_ESM.docx]

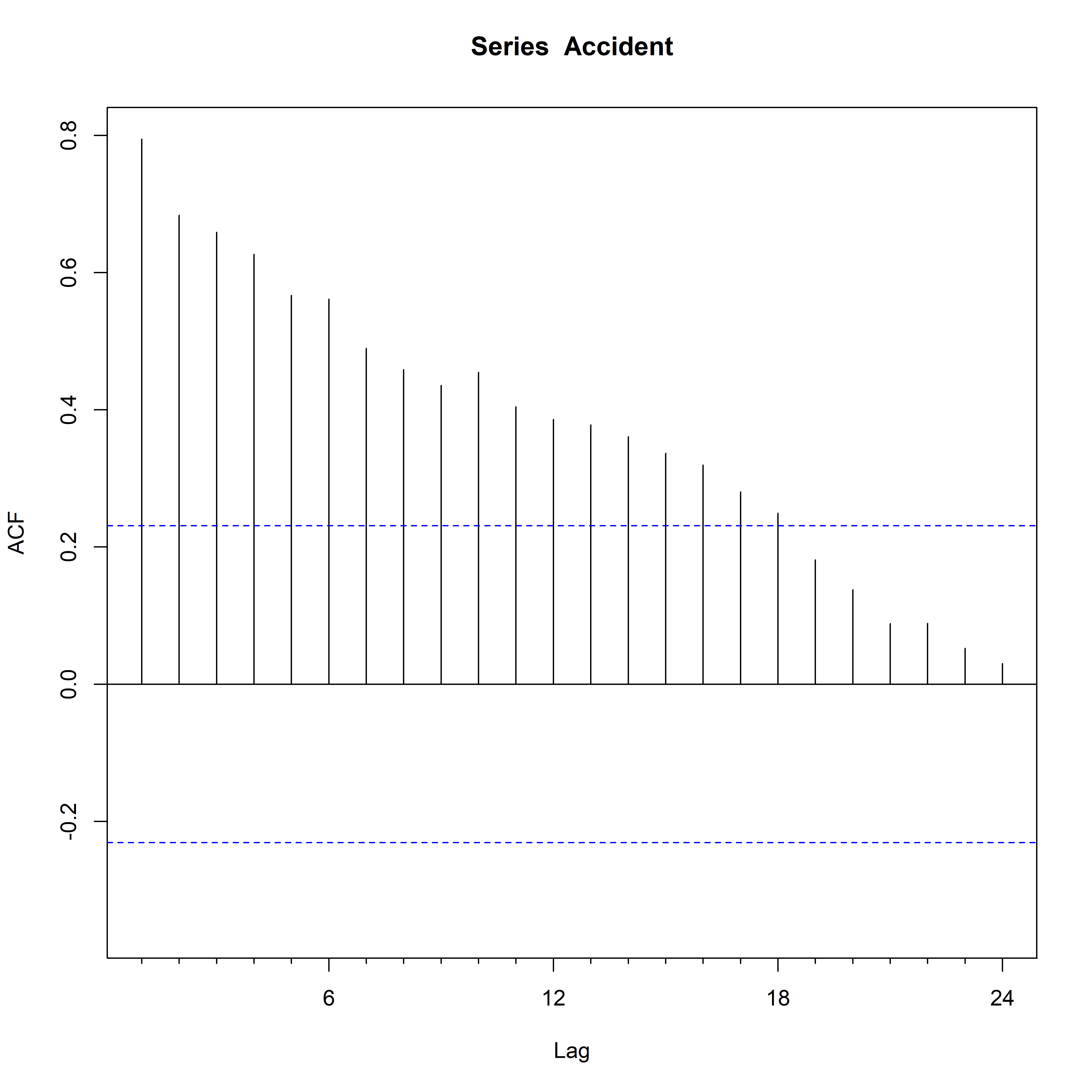


Fig S1. ACF of the Number of Accidents


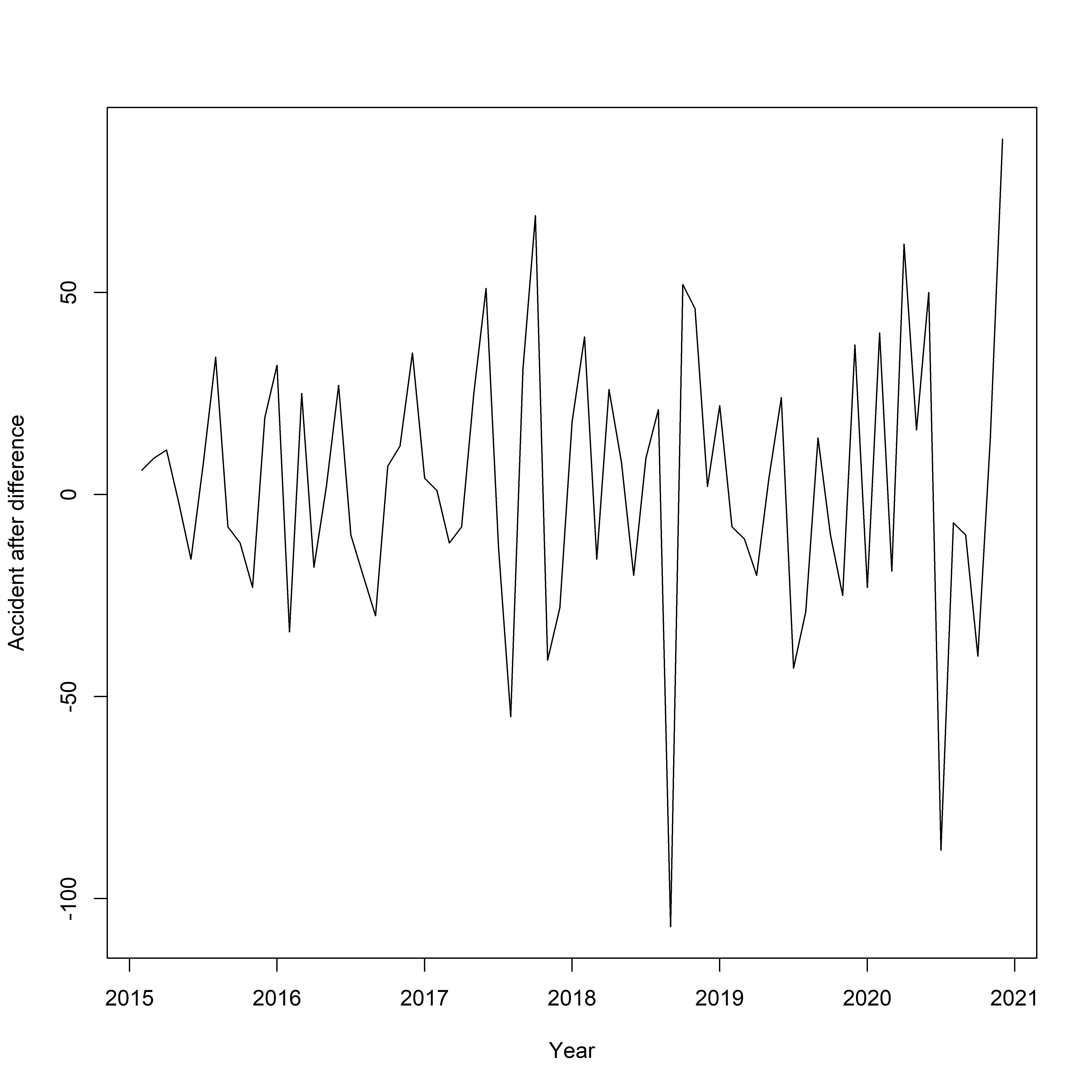


Fig. S2. Time Series Plots of the First Difference the Number Accidents

| 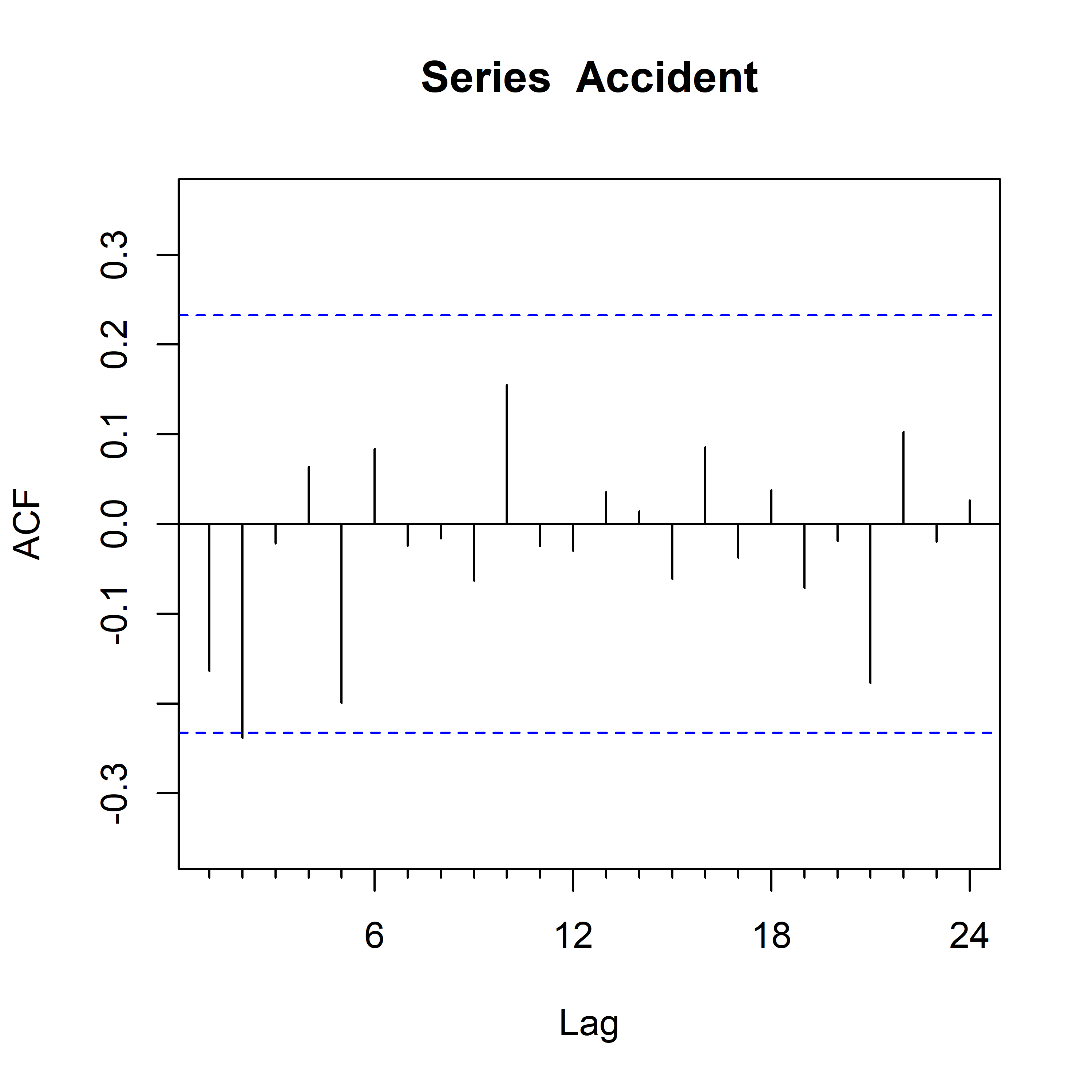  a | 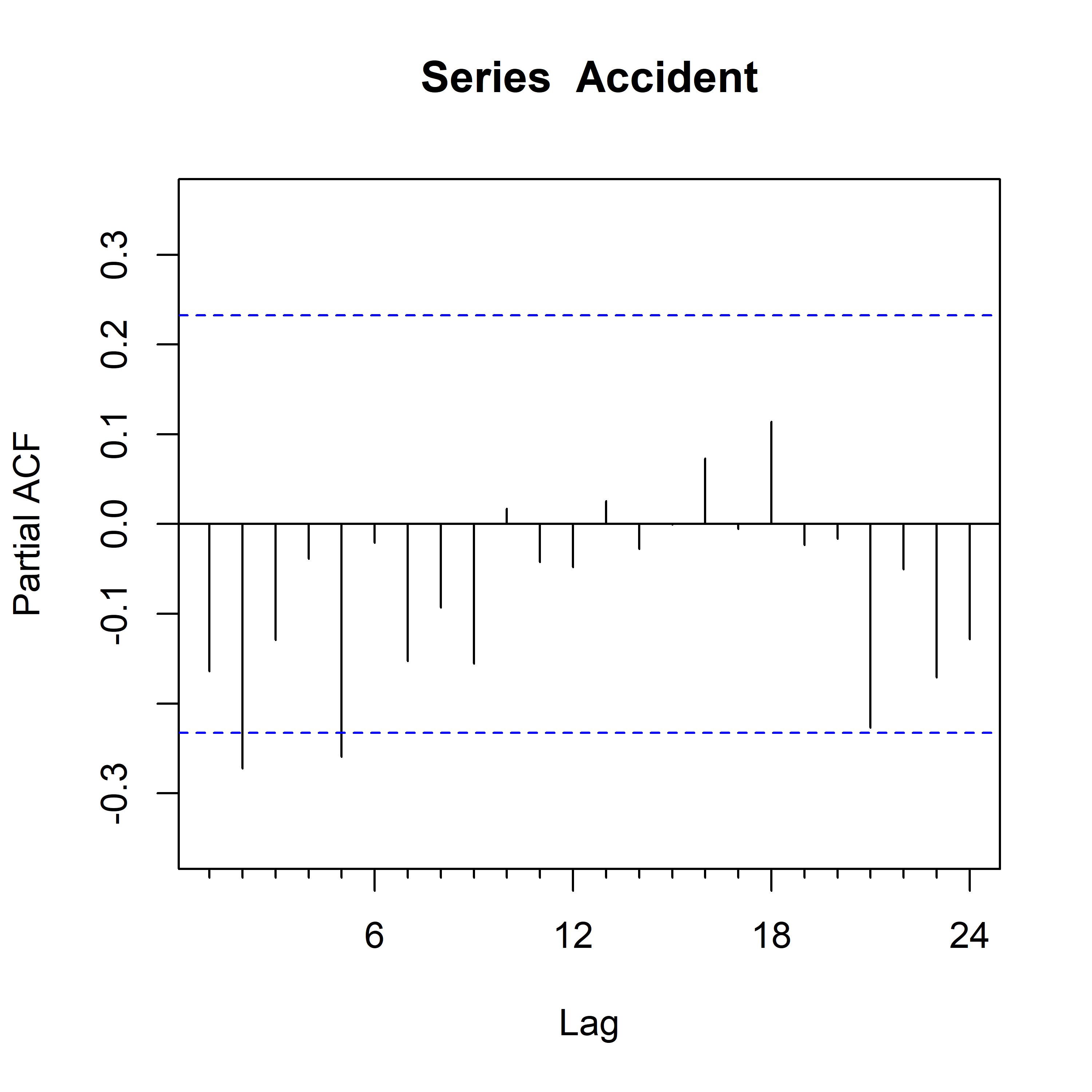  b |
| --- | --- |

Figure S3.a: ACF of the First Difference of the Number of Accidents, b: PACF of the First Difference of the Number of Accidents

Table S1. Number of accidents predicted using the final ARIMAX model in the 12 months of (21March 2022- 20 March 2023)

| Year | Month | Number of predicted accidents | 95% Confidence interval for the number of predicted accidents | |
| --- | --- | --- | --- | --- |
|  |  |  | Minimum | Maximum |
| (21March 2022- 20 March 2023) | March | 192 | 134 | 250 |
|  | April | 227 | 164 | 289 |
|  | May | 229 | 162 | 295 |
|  | June | 291 | 224 | 357 |
|  | July | 307 | 240 | 373 |
|  | August | 357 | 290 | 423 |
|  | September | 269 | 202 | 335 |
|  | October | 262 | 195 | 328 |
|  | November | 252 | 185 | 318 |
|  | December | 212 | 145 | 278 |
|  | January | 226 | 159 | 292 |
|  | February | 314 | 247 | 380 |


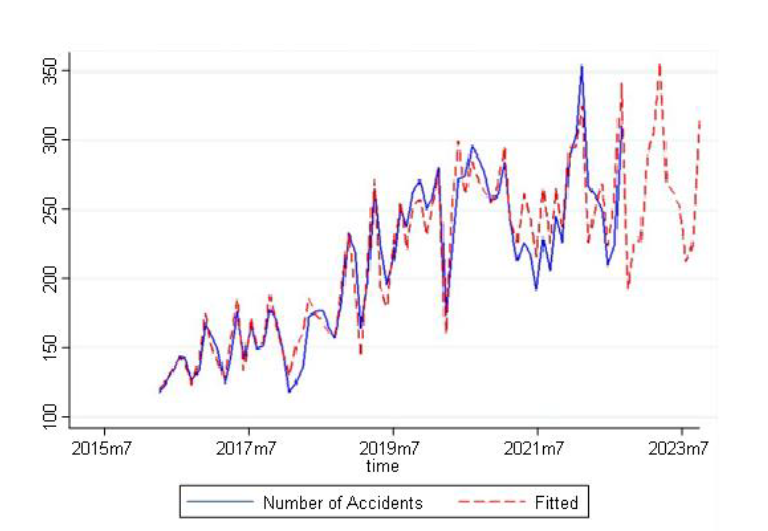


Fig. S4. ARIMAX Model Forecast for the Number of Accidents
